# Hearing screening for healthy aging: the U.K. should take a targeted approach

**DOI:** 10.1093/geront/gnaf295

**Published:** 2025-12-10

**Authors:** Jack Stancel-Lewis, Tom Dening, Eithne Heffernan, Adrian C Davis, Helen Henshaw

**Affiliations:** Nottingham University Business School, University of Nottingham, Nottingham, United Kingdom; Mental Health and Clinical Neurosciences, School of Medicine, University of Nottingham, Nottingham, United Kingdom; Mental Health and Clinical Neurosciences, School of Medicine, University of Nottingham, Nottingham, United Kingdom; School of Sport, Exercise & Health Sciences, Loughborough University, Loughborough, United Kingdom; AD Cave Solutions Limited, Stroud, Gloucestershire, United Kingdom; Mental Health and Clinical Neurosciences, School of Medicine, University of Nottingham, Nottingham, United Kingdom; National Institute for Health & Care Research (NIHR), Nottingham Biomedical Research Centre, Nottingham, United Kingdom

**Keywords:** Hearing (loss), Dementia, Disparities, Health Economics, Screening

## Abstract

Hearing loss affects an estimated 18 million U.K. adults and is the third leading cause of years lived with disability worldwide. Hearing loss diminishes quality of life, activities of daily living, mental health, and negatively impacts the economy. When left unaddressed, hearing loss exacerbates health inequalities and is associated with an increased risk of developing dementia. Hearing loss and dementia frequently co-occur and have overlapping risk factors. People from disadvantaged backgrounds typically access hearing and memory services at a more progressed stage of their conditions. Early intervention is essential to mitigate negative effects and reduce disease burden. Universal adult hearing screening is not currently recommended by the U.K. National Screening Committee, despite evidence of cost-effectiveness and international recommendations. We argue that the U.K. should adopt targeted, risk-stratified screening that prioritizes those at greatest risk of dementia and people in deprived communities, where unmet need is highest. Such an approach is practical, equitable, and consistent with the Governments’ prevention and personalized care agenda set out in the 10-year Health Plan for England. We outline the evidence, discuss implementation pathways and targeting strategies, and call on policymakers to commission a targeted, risk-stratified adult hearing screening pilot. A successful program would address unmet need, reduce inequalities, and further inform global debates on hearing screening strategies.

## Introduction: why this debate matters

Hearing loss is one of the most common and disabling conditions in later life. In the U.K., 18 million adults, which equates to one in three, live with some degree of hearing loss (Akeroyd & Munro, 2024). Hearing loss is the third largest contributor to years lived with disability (YLD) behind lower back pain and migraine (Haile et al., 2021).

Hearing aids are effective at improving hearing and health related quality of life and listening ability in adults with moderate hearing loss (Ferguson et al., 2017). Yet, only one in five of those who could benefit from hearing aids have them (Dawes et al., 2014) and it takes an average of 10–12 years for a person to seek help for hearing difficulties (Davis et al., 2007).

Age- and lifestyle-related hearing loss generally progresses slowly and often goes undiagnosed. In the U.K., it has been reported that only 38.1% of people who acknowledged a hearing loss sought help from their family doctor (General Practioner - GP). Among them, one in five were not referred for hearing assessment. However, when a referral was made, most patients (78.9%) were willing to try hearing aids (Tsimpida et al., 2024).

When left unaddressed, hearing loss limits access to spoken language and environmental cues, negatively impacts activities of daily living, and increases the risk of social isolation, depression, falls, cognitive decline and dementia (Ferguson et al., 2017). Hearing loss costs the U.K. economy £25.5 billion each year: £9 billion in lost productivity and £16.5 billion associated with poorer quality of life (Shield, 2019). Globally, every $1 spent on hearing care results in a return on investment (ROI) of $15 when considering healthcare delivery and a societal perspective (Tordrup et al., 2022). Thus, a 90% scale up in community hearing care globally could avert 130 million disability-adjusted-life-years (7-year horizon) translating to $1.3 trillion in gains (Tordrup et al., 2022).

Despite this burden and opportunity, there is no systematic adult hearing screening program in the U.K. Adult hearing screening is recommended by The World Health Organization (WHO) as part of their Integrated care for older people apporach guidelines (WHO, 2024). The WHO consider adult universal hearing screening as cost effective; their World Report on Hearing details that for every $1 invested, an ROI of $1.62 is achieved in high-income countries (WHO, 2021b). However, the U.K. National Screening Committee (NSC) has repeatedly decided not to recommend universal hearing screening in adults.

This article argues that targeted and risk-stratified screening, rather than universal approaches, represents a more feasible, cost-effective, and equitable path forward. We examine the evidence base, outline potential implementation models, and make a policy call for the conditional adoption following successful piloting and equity analysis in the U.K.

## Why universal screening currently falls short

The case for universal screening appears strong; hearing aids are highly effective at improving quality of life, earlier intervention is cost-effective, and international bodies endorse this approach. Key modeling studies demonstrate incremental cost-effectiveness ratios (ICERs) far below the National Institute for Health and Care Excellence (NICE) ICER limit of £20,000–£30,000 per quality adjusted life year (QALY) (National Institute for Health and Care Excellence, 2022).

In the U.K., Davis et al. (2007) examined the cost-effectiveness of hearing screening in adults aged 55 years and older. They identified that hearing screening and the provision of amplification (compared to standard care), provided nine additional years of benefit, over a life time horizon, resulted in an ICER of £1,076 per QALY using the Health Utility Index (HUI) and £2,269 per QALY using the six-dimensional health state short form of health-related quality of life, using a cut off of 35 dB HL in the better ear, reporting that 95% of people would accept and wear hearing aids at this level.

Morris et al. (2013) compared hearing screening in primary care to the current GP referral model. They suggested that a one-stage pure-tone screening test at age 60, in both ears, with a cut off of 35 dB HL, resulted in an ICER of £1,461 per QALY over a 25-year time horizon. This suggests that hearing screening in adults is cost-effective. More recently, a NICE cost-utility analysis found that earlier provision of hearing aids at age 65, was more cost-effective than delayed provision (10 years) in the general population, with an ICER of £3,976 per QALY gained over a lifetime horizon.

Table 1 highlights this evidence, including inflation adjusted cost per QALY, suggesting that inflated costs are still within NICE limits. (Table 1).

**Table 1.** Evidence on cost-effectiveness of adult hearing screening, including inflation adjusted cost per QALY.

Inflation adjusted QALY							
Study	Objective	Population	Cost/QALY	Base year CPI index (year of publication)	Inflation factor	Adjusted Cost/QALY to 2024	Implication
**Screening or earlier intervention to address hearing loss**							
**Davis et al. (2007) (HUI)**	Population hearing screening vs. usual care	Adults aged 55–74 years	£1,076	81.8	1.64	£1,765	Universal screening judged cost-effective
**Davis et al. (2007) (SF-6D)**	Population hearing screening vs. usual care	Adults aged 55–74 years	£2,269	81.8	1.64	£3,721	Universal screening judged cost-effective
**Morris et al. (2013)**	Population screening in primary care vs. usual care	Adults aged 60 years	£1,461	96.1	1.39	£2,031	Universal screening judged cost-effective
**Earlier intervention to address hearing loss**							
**NICE (2018)**	Earlier intervention (10 years) through current care pathways	Adults age 65 years	£3,976	105.9	1.26	£5,010	It is cost effective to provide hearing aids 10 years earlier than to wait.
**Regular hearing assessment to address hearing loss in MCI or dementia**							
**NICE (2018)**	Bi-annual opportunistic (re)assessment for people with MCI or dementia	Adults with MCI or dementia	£14,337	105.9	1.26	£18,065	It costs more to wait and screen those with dementia bi-annually than to implement screening and earlier intervention
**Screening to address hearing loss and cognitive decline or dementia risk**							
**Mukadam et al. (2020)**	Screening to prevent 3.3% of dementia cases through hearing loss intervention	Middle aged adults aged 45–65 years	−£607 (dominates)	108.7	1.23	−£747 (dominates^a^)	Screening and treating hearing loss results in significant health and economic benefits

2024 CPI (133.9)/CPI publication year = inflation factor (Turner et al., 2019).

Inflation factor x cost per QALY = Adjusted cost/QALY to 2024 (Turner et al., 2019).

Consumer Price Inflation (CPI) index (MM23) data were extracted and averages for the year used (CPI base year 2015 = 100) (Office for National Statistics, 2025).

a if a QALY dominates it is cost saving.

Abbreviations: QALY = quality adjusted life years, MCI = mild cognitive impairment, NICE = National Institute for Health and Care Excellence, CPI = consumer price index, SF-6D = Soft-Form 6-Dimentions, HUI = health utilities index.

Yet, hearing screening is currently not recommended by the Department of Health and Social Care’s UK NSC, who make decisions on the commissioning of U.K. screening programs. They decline universal screening largely on practical, rather than cost grounds. Following the most recent review published in 2021, the NSC concluded that systematic population screening for hearing loss in adults did not meet their criteria for recommendation. While they acknowledged that hearing aids are effective at treating hearing loss, there was insufficient evidence of improved outcomes from earlier intervention because of screening, it was not clear if patients are willing to wear hearing aids, how well managed hearing loss is currently in the U.K. how to implement hearing screening, and whether new testing techniques were sufficiently sensitive and specific (The UK National Screening Committee, 2021). The next review is estimated to be completed in 2025.

A significant challenge associated with delivering universal hearing screening is the impact this undertaking would have on the health system. NICE suggested in 2023 it may cost £20.7 million a year (should new innovative commissioning models not be developed) to refer the additional 23% of patients for audiology assessment that present with hearing difficulties to the GP for the first time, (NICE, 2023b). The resource implications, in terms of service provision, training, personnel to deliver the screen, the increase in activity for audiology services, and the total cost and therefore opportunity cost may make universal screening unsustainable, unless there is a significant shift in the way services are delivered.

Adult hearing screening pilot initiatives have been implemented across several European countries, including Italy (Paglialonga et al., 2011), Cyprus (Thodi et al., 2011), and the Netherlands indicating cost effectiveness, acceptability and feasibility (Linssen et al., 2015). However, these programs have primarily focused on age, rather than employing stratified approaches that consider additional risk factors.

## The case for targeted, risk-stratified screening

### Dementia link

Livingston et al. (2024) report that optimal management of hearing loss during midlife could potentially result in 7% fewer cases of dementia at the population level. This statistic is derived by calculating the population attributable fraction, which accounts for the prevalence of a factor, like hearing loss, in a specific population and the relative risk it poses for developing a condition like dementia. The study performed a random effects metanalysis of six studies that met quality criteria and reported an almost 40% increased relative risk of developing dementia (RR (95% confidence interval [CI]) = 1·37 (1.00–1.87)). The mean age of participants included at baseline was 59, with a follow-up period range between 6 and 12 years, each study used an objective measure of hearing and incident dementia as the outcome. All six studies made adjustment for cardiovascular risk factors, cognitive or education at baseline and age as covariates. However, some studies had limited ethnic diversity, therefore external validity and generalizability may be impacted. There was a high degree of heterogeneity (80%) suggesting large variation between the study outcomes. The CI includes 1.00, suggesting the finding is on the borderline of significance and should be interpreted with some caution. There are some known risk factors that are not adjusted for in all studies, increasing the risk that the association may be due to other shared risk factors, and due to the observational nature of the studies no direct causal relationship has yet been established.

Nevertheless, high quality evidence does exist for the use of hearing aids changing the trajectory of cognitive decline. A systematic review and meta-analysis of the effect of restorative hearing devices (e.g., hearing aids, cochlear implants) on the risk of cognitive decline in a pooled cohort (126,903 participants) from eight observational studies with a follow-up period between 2 and 25 years showed that using hearing aids could reduce the risk hazard of cognitive decline by as much as 19% (HR [95% CI] = 0.81 [0.81–0.87]) and meta-analysis in 11 studies showed an increase in cognitive test performance by 3% (ratio of means [95% CI] = 1.03 [1.02–1.04]) (Yeo et al., 2023). The Aging and Cognitive Health Evaluation in Elders (ACHIEVE) randomized controlled trial showed that hearing aids reduced cognitive decline by as much as 50% over a 3-year period in older adults at higher risk of cognitive decline (familial history, comorbidities) (Lin et al., 2023). More recently, a study of individuals at risk of cognitive decline in the Atherosclerosis Risk in Communities (ARIC) cohort used in the ACHIEVE trial showed that 8-year incident dementia could be attributable to audiometric hearing loss (Ishak et al., 2025), and that treating hearing loss might delay dementia for a large number of older adults. This is further supported by a U.K.-based pilot RCT examining the provision of hearing aids for people diagnosed with mild cognitive impairment (MCI), with findings showing a signal of efficacy, acceptability of the intervention and feasibility of a full-scale RCT (Yu et al., 2025). Finally, evidence from a 20-year follow up in the Framingham Heart Study highlights that early intervention (before the age of 70 years) is key for reducing dementia risk (Francis et al., 2025).

The annual cost of dementia to the U.K. economy is £34.7 billion, set to rise to £94.1 billion by 2040 (Alzheimer’s Society, 2020). There is a high prevalence of hearing loss in people who live with dementia (Nirmalasari et al., 2017; Ray et al., 2019) and their care should be considered as important as those at risk of the condition. Untreated hearing loss can exacerbate dementia related behaviors, such as confusion and agitation, and may result in greater care needs (Haque et al., 2012; Mamo et al., 2017). Appropriate management of hearing loss has been shown to increase hearing related quality of life (Wolff et al., 2024) and health utility (Borre et al., 2023), including in people with dementia (Leroi et al., 2020; Mamo et al., 2017).

NICE (2018) recommends that people who have been diagnosed with MCI and dementia should be referred for audiological (re)assessment every 2 years. Yet it is unclear how often this guidance is implemented. Evidence suggests that diagnosis of hearing loss significantly declines following diagnosis of Alzheimer’s disease (Hokkinen et al., 2025). When hearing loss is diagnosed, hearing aids are underutilized in people with cognitive decline and dementia (Nirmalasari et al., 2017). However, earlier intervention with hearing aids, prior to initial stages, or during earlier stages of decline results in greater adaptation to, and use of hearing aids (Naylor et al., 2022). Therefore, supporting earlier detection and intervention may improve quality of life, reduce the risk of exacerbating dementia related symptoms and may ultimately support people to live independently for longer (Leroi et al., 2020). A NICE cost-utility analysis found that earlier provision of hearing aids was more cost-effective than delayed provision in the general population; sensitivity analyses assessing the cost-effectiveness of referring people with dementia and MCI for audiology assessment every 2 years, resulted in an ICER of £14,337 per QALY over a lifetime horizon, discounted at 3.5% a year (NICE, 2018).

Brent (2019) performed a *cost–benefit* analysis estimating direct utility benefits from hearing aids and indirect utility benefits from the reduction of dementia symptoms arising from the use of hearing aids over a 23-year time horizon, and despite the high cost of hearing aids in the United States (>$8,000), the analysis identified a benefit–cost ratio (BCR) of 29.23, with sensitivity analysis BCR of 20.46, 18.42, and 6.31 based on increasing cost estimates. These findings support the early and sustained use of hearing aids, particularly in populations at risk of cognitive decline.

Screening hearing in those at risk of dementia is a pressing unmet need for patients, carers, and clinicians, highlighted as a top priority by a recent James Lind Alliance priority setting partnership (Heffernan et al., 2025). Mukadam et al. (2020) conducted a cost-effectiveness analysis modelled on screening hearing in adults (aged 45–65 years) and providing hearing aids to 8% of the middle-aged English population. They reported a QALY gain of 0.0798 and a discounted lifetime costs saving of £607 per person attributable to reducing dementia related care costs. They highlighted an annual cost to the NHS of £335 million; however, modeled a net cost saving of £776 million per year, with a reduction in dementia diagnoses of 3.3%.

It is important to acknowledge that the economic study by Brent (2019) and Mukadam et al. (2020) make assumptions that hearing aids can improve dementia symptoms and prevent dementia cases, respectively. Although not all studies show significant effects, the convergence of evidence suggests that hearing intervention can alter cognitive decline trajectories, thus delaying (but not necessarily eliminating) dementia risk, particularly when delivered early. Importantly, linking hearing loss and dementia may increase the stigma associated with hearing loss or induce anxiety associated with screening outcome, and therefore may result in people further avoiding help-seeking (Dawes & Munro, 2024). Consequently, appropriate protocols and framing of why people are being invited for screening needs to be based on hearing loss risk and hearing as part of healthy aging, in turn normalizing hearing screening and intervention.

### Structural and demographic risk factors

Age remains the strongest predictor of hearing loss in the U.K. (Akeroyd & Munro, 2024), with >50% of those aged 55 years and older and 80% aged 70 years and older affected (RNID, 2024). Studies have identified significant disparities between different ethnic groups and hearing loss prevalence and the use of hearing healthcare (Scholes et al., 2018). People from ethnic minority backgrounds were highlighted to be around three times more likely to have hearing loss than people from a White British background (Dawes et al., 2014). People from ethnic minority and migrant groups are also more likely to perform poorly on speech-based screening tests (Taylor et al., 2020) and people from ethnic minority groups, especially Black people and people from the Indian ethnic group, are significantly less likely to use hearing aids, even when they report similar levels of hearing difficulty (Taylor et al., 2023). In England, hearing loss prevalence is highest in the 20% most deprived communities; people with manual jobs, those with lower income, and individuals with no formal qualifications are at greatest risk (Tsimpida et al., 2019). Men in the lowest socioeconomic groups exhibit the highest burden but have significantly lower rates of hearing aid use (Scholes et al., 2018). In addition, a pronounced north–south divide exists, with age‑adjusted hearing loss prevalence among adults aged 71–80 years ranging from 36% in the South East to 49% in the North East of England (Tsimpida et al., 2023). The relationship between deprivation and hearing loss can act as a vicious cycle, each increasing the risk of the other (Tsimpida et al., 2021). Therefore, timely identification and management of hearing loss is crucial to promote equity, healthy aging, improve quality of life, and maintain independence (World Health Organization, 2021b).

Black people in the U.K. have a higher incidence of dementia, and both Black and South Asian people with dementia die at a younger age than White individuals (Mukadam et al., 2023). People from South Asian and Black backgrounds with dementia face inequity in diagnostic rates, poorer treatment outcomes and a lack of culturally specific support (Subramaniam & Mukaetova Ladinska, 2025). The risk of dementia is higher among individuals in more deprived U.K. communities; in one U.K. study people with low socioeconomic status had an HR of 1.43 (CI 1.09–1.86), indicating approximately 40% greater risk of developing dementia (Wang et al., 2024). This relationship is partly driven by social factors (e.g., lower educational attainment, social isolation) lifestyle (e.g., poor diet, smoking, alcohol use), environmental exposures (e.g., pollution), and is further compounded by poorer health, in particular, depression, cardiovascular risk factors such as hypertension, hyperlipidemia (influenced by the presence of apolipoprotein E (APOE) ε4 gene), and diabetes (Ou et al., 2024; Wang et al., 2024). These factors have also been shown to increase the risk of hearing loss. Hearing loss risk is increased by smoking, noise exposure, and cardiovascular risks (Agrawal et al., 2008), and risk of hyperlipidemia with statin use as a proxy (Nash et al., 2011).

Melis et al. (2023) developed the U.K. Biobank Dementia Risk Score (UKBDRS), for predicting up to 14-year all cause dementia among individuals aged 50–73 years. The model adopts a range of factors, including age, education, deprivation, diabetes, depression, stroke, parental history of dementia, and presence of APOE ε4. The risk predictor outperformed three other commonly used scores: the Australian National University Alzheimer’s Disease Risk Index, the Cardiovascular Risk Factors, Ageing, and Dementia score (CAIDE), and the Dementia Risk Score (DRS). However, databases such as the U.K. Biobank tend to have limited sociodemographic profiles, including being male dominated. Furthermore, evidence indicates that the predictive validity of certain dementia risk scores (e.g., CAIDE) may vary across populations and age groups (Stubbs et al., 2025) therefore a combination of risk-scores and further validation with datasets that have greater representation of the population is required. By capturing vast datasets and training new models, machine learning could support the continuous improvement and refinement of risk score tools. This approach is appealing, because many of the risk factors for dementia, are also risk factors for hearing loss.

Deprivation is a key factor in these risk models, a risk for both dementia and hearing loss, and a proxy for poor cardiovascular health. Thus, hearing screening could be offered to those who live in the poorest communities first, aligning with the NHS’s Core20PLUS5 policy to tackle healthcare inequalities supporting the most deprived 20% of society, local priority groups (e.g., ethnic minority populations, inclusion health groups) and the five priority clinical conditions that require accelerated improvement that includes cardiovascular disease (CVD), which could act as a proxy for both hearing loss risk and dementia risk (NHS England, 2023). This will ensure more equitable care can be provided in a targeted way and help avoid widening inequalities associated with both hearing loss and dementia, aligning with the government’s strategy to advance preventative and personalized care. Hearing loss prevalence was identified as highest in the most deprived quintile of the population, with a clear social gradient (Tsimpida et al., 2020), suggesting a socio-spatial approach would be appropriate, and supported by large population-based studies. Audiology service coverage would need to be adapted to adjust for increase in demand via referrals in these areas, ensure culturally appropriate support and messaging, and align with policy to move care into the community.

### Implementation

**Figure 1.**
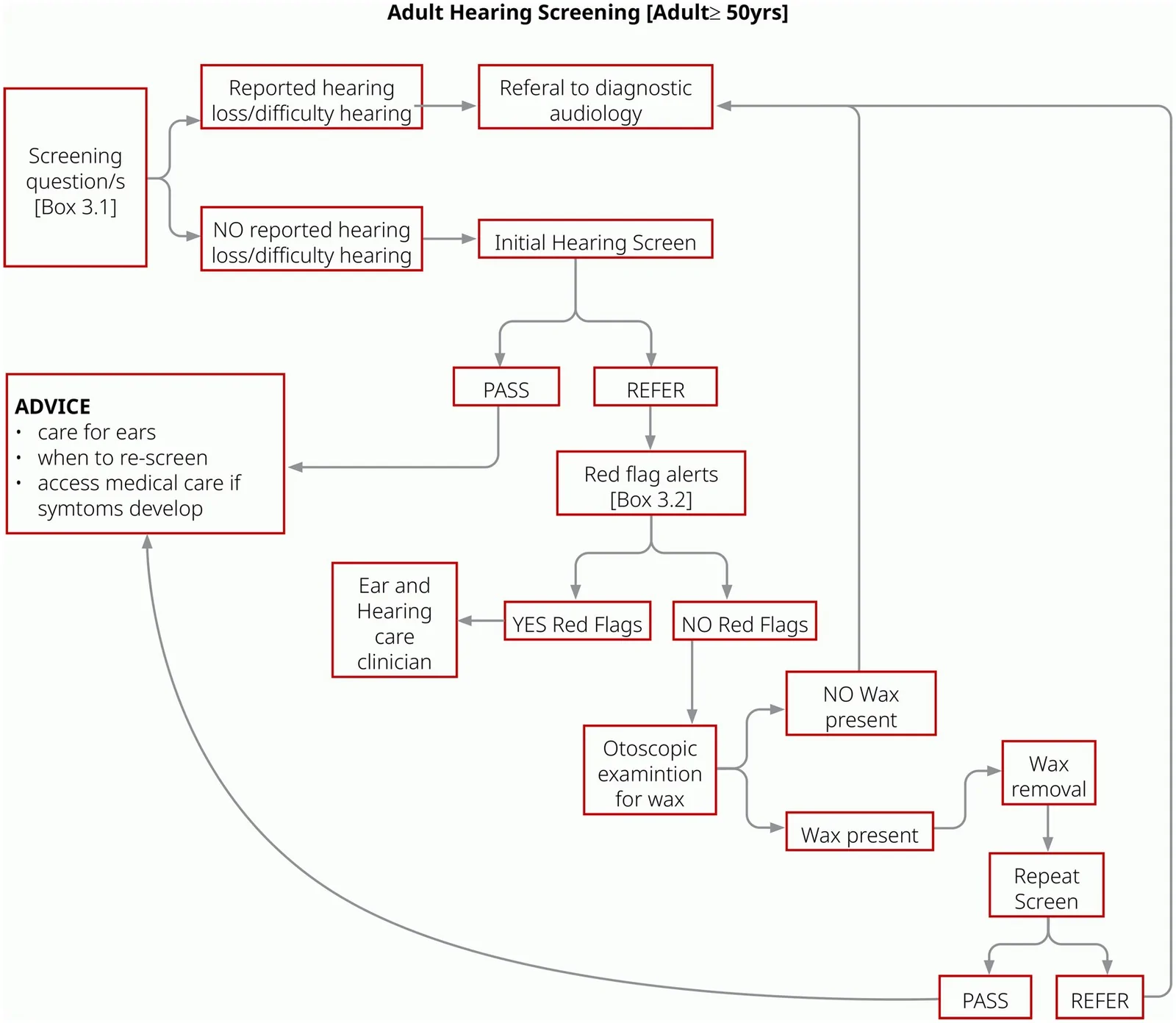
Steps in hearing screening and follow-up of older people. From World Health Organization (2021). *Hearing screening: considerations for implementation*. Geneva: World Health Organization, p. 41. Licence: CC BY-NC-SA 3.0 IGO.

The WHO recommend three screening hearing tests in adults; these include presenting pure tones in both ears at a fixed decibel level, the digits-in-noise test, and the determination of air conduction thresholds through pure-tone threshold screening (World Health Organization, 2021a). These tests can be delivered in clinical, community, and residential settings, or remotely. The WHO set out clear recommendations for the implementation of hearing screening procedures, see Figure 1.

The harms of screening hearing are considered to be low, however, there is a risk of increased anxiety, and therefore appropriate messaging is important (Jin, 2021). By selecting a cut-off of 35 dB HL, the risk of overdiagnosis of hearing loss is minimized, and would reduce the burden placed on healthcare services, with the estimated hearing loss prevalence dropping from 18 million to 4.6 million adults (Akeroyd & Munro, 2024).

Digital screening tests, such as digits-in-noise, can provide a rapid 2-min screen that are easily implemented online or through the WHO HearWHO app (World Health Organization, 2021a), with no need for previous experience, specialist training, or administration by a professional. However, screening tests can also be guided by a healthcare professional or support worker, who can ensure that environmental conditions are suitable (Swanepoel, 2020; Swanepoel et al., 2019).

Dawes et al. (2021) assessed the feasibility of internet-delivered U.K. adult hearing screening using the digits-in-noise test and estimated they reached 70% of the target population via postal invite, concluding that the approach offers potential large scale early identification of hearing problems that is both acceptable and equitable. Screening tests could be automated, self-administered, and enhanced by artificial intelligence. Yet, to justify their implementation strong evidence and a considered approach would be required regarding their feasibility and acceptability for diverse populations and individual needs. Other issues such as occlusive ear wax, would need to be managed, as well as looking at ways to provide personalized, culturally appropriate care.

New delivery models are already emerging; a pilot delivered in community pharmacy using a smartphone-based screening tool successfully managed thousands of patients, reducing pressure on GPs, Ear, Nose and Throat (ENT) clinics and minimized inappropriate referrals to audiology (NHS South Central and West Commissioning Support Unit, 2024). The approach included otoscopic examination, wax removal when required and a validated pure tone hearing screen using the TympaHealth smartphone device. A total of 7,648 patients were referred; 85% received an ear canal examination followed by earwax microsuction and/or hearing check, and 193 were referred to secondary care (NHS South Central and West Commissioning Support Unit, 2024). The pathway was both feasible and acceptable to patients and clinicians, highlighting the opportunity to use this novel approach to hearing screening in the community, reducing the burden on GPs and audiology services. This approach could be adapted to meet local resource availability and replicated in other community settings like community diagnostic centers or within primary care settings, by a range of healthcare personnel including audiologists.

As a first step, Alzheimer’s Research UK (2023) recommend incorporating a hearing screen within the NHS health check. This could help tackle health inequalities associated with dementia risk, by offering a check to adults aged 40–74 years, including those less likely to seek help for hearing difficulties. To ensure acceptability and sustainability, hearing screening would need to be delivered in a targeted manner. As the NHS Health Check is designed to assess CVD risk and includes a check related to dementia risk for individuals aged 65 and older (NHS, 2023), hearing screening could be offered to those identified as at high risk during these assessments. This approach would also help ensure that performance on memory tasks is not adversely affected by hearing impairments, and the most appropriate management approach can be implemented.

Focusing purely on peripheral hearing loss may miss a vital opportunity. Marinelli et al. (2022) suggest that informant-reported hearing difficulties resulted in greater association with the diagnosis of dementia, compared to PTA. This supports the viewpoint that central auditory processing may be an important early indicator of incipient dementia and manifest as communication difficulties (Levett et al., 2025). Utilizing central auditory tests could act as both a measure for auditory function, and an early indicator for dementia.

Memory services rarely assess hearing systematically. In the UK, the Memory Service National Accreditation Programme quality standards include asking about comorbid sensory issues during memory assessment. However, the 2023/2024 national memory assessment service dementia audit highlighted that only 63% of patients were asked about their hearing, a mere 5% increase from 2021 (Royal College of Psychiatrists, 2022, 2024). This is despite NICE recommending regular audiology checks for people with dementia or mild cognitive impairment (NICE, 2018). Embedding screening in such pathways could improve detection and support fairer access to care.

**Figure 2.**
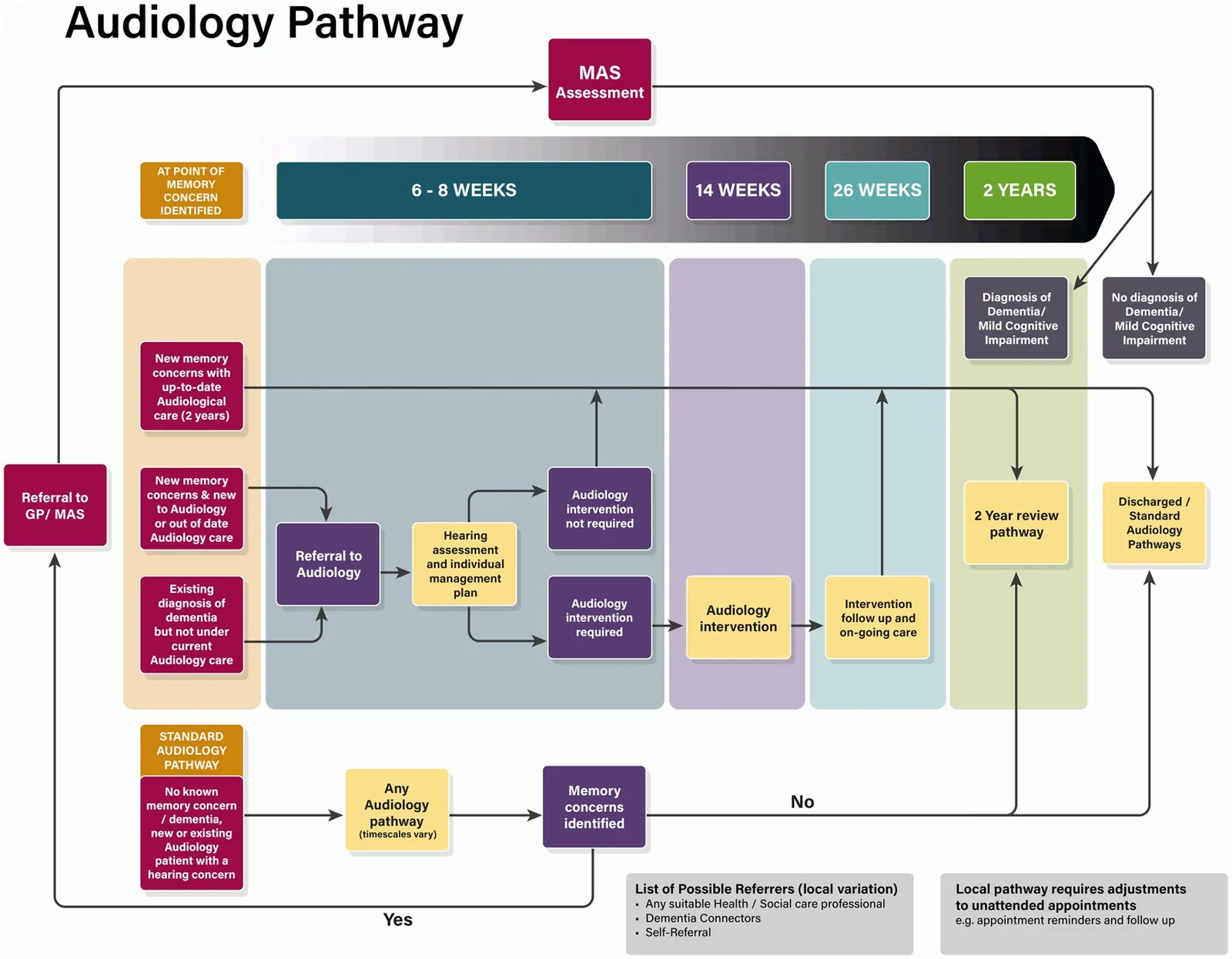
All Wales Dementia Hearing Loss Pathway. From NHS Wales Performance and Improvement (2025). *All Wales dementia hearing loss pathway*. https://performanceandimprovement.nhs.wales/functions/quality-safety-and-improvement/improvement/our-work/mental-health/dementia-care/all-wales-dementia-hearing-loss-pathway/, p. 9. Licence: CC BY-NC-ND 4.0.

In Wales, U.K., hearing assessments are integrated into memory assessments services and dementia reviews. The “All-Wales Dementia Care Pathway” published by NHS Wales Performance and Improvement (2025) in May, outlines a comprehensive and person-centered approach to managing audiological needs in individuals with memory concerns or a dementia diagnosis, see Figure 2. Initial roll out of the pathway resulted in 97% of new patients referred to audiology being diagnosed with a hearing loss with 84% uptake of hearing aids. The pathway supports proactive identification of hearing loss as part of cognitive assessments and follow-up reviews aligning with NICE recommendations for people with dementia and MCI. This demonstrates how targeted hearing screening can be implemented and integrated effectively within existing service structures, supporting the broader goals of dementia prevention and care equity. If this approach was to be rolled out across England and Scotland, a key focus would need to be on training the audiology workforce to support people with dementia, and ensure all services are dementia friendly.

Yet, memory assessment clinics may be considered as addressing the issue too late in terms of cognitive status. Earlier hearing screening in people at high risk of dementia, at the latter stages of midlife, for example, starting at age 55 years, would offer potential for greater benefits (Livingston et al., 2024). An alternative approach would be to identify the target screening population by selecting people with numerous risk factors or using validated dementia risk scores in primary care settings. A similar approach is already in use to assess cardiovascular risk; QRISK3 is recommended by NICE and calculates a patients 10-year risk of CVD/event using routinely collected patient data such as age, ethnicity, smoking status, systolic blood pressure, body mass index, lipid profile, postcode (a proxy for deprivation), and other cardiovascular risk markers to generate a risk score (NICE, 2023a). QRISK3 tool can be embedded within electronic health record systems so that calculations are automated. Dementia risk prediction tools like the UKBDRS and CAIDE are not currently used for the purpose of risk reduction but are widely used in research and clinical trials (Anstey et al., 2021). Risk-score tools could be further developed and refined for embedding into primary care patient records, thus automating the identification of individuals at high risk and offering a hearing screen and early intervention as part of a broader prevention and healthy aging strategy. Yet, the choice of risk scores will be critical and should be piloted.

These examples demonstrate that targeted hearing screening can be successfully integrated within existing care pathways, while supporting early identification and equitable access for populations at greatest risk. To translate this into broader practice, a structured approach is required that considers which screening tools to use, where and how screening is delivered, and which populations should be prioritized.

## Call to conditionally adopt targeted, risk-stratified hearing screening in the U.K.

Hearing loss is a major public health issue that undermines healthy aging, increases health inequalities, and is closely intertwined with dementia risk. As our review of the evidence has shown, universal screening, although cost-effective in principle, faces substantial feasibility and resource barriers that have contributed to the UK NSC to withhold recommendation. At the same time, growing evidence demonstrates that earlier intervention, particularly in those at high risk of dementia or in deprived communities, can yield significant health, social, and economic benefits. Table 2 summarizes key elements for the design and implementation of targeted adult hearing screening in the U.K., highlighting practical options and recommendations for design and piloting.

**Table 2.** Key elements for implementation of targeted and risk-stratified adult hearing screening in the U.K.

Dimension	Option	Details	Recommendation
**Screening tools**	Pure tone audiometry (PTA) screening	Handheld devices such as HearCheck and TympaHealth can provide a rapid and accurate threshold screen, with the latter offering additional capability for wax removal.	Combine and pilot both approaches
	Digits-in-noise	Speech-in-noise test with ease of delivery online or via an app. Dichotic versions can reveal ear asymmetries or central auditory deficits.	
**Settings**	Community Pharmacy	Accessible community settings, low stigma. May enable ­accessibility to underserved communities including ethnic minority groups	Allow flexibility in adoption to suit local context and resource
	General Practice	Primary care setting, enabling integration with NHS Health Checks and/or dementia risk assessment pathways	
	Neighbourhood health / Community diagnostic centers	Multimodal testing centers in community settings	
**Targeting / stratification approach**	Candidate risk scores for dementia/­cardiovascular disease	E.g., UK Biobank Dementia Risk Score (UKBDRS); Cardiovascular Risk Factors, Ageing, and Dementia score (CAIDE); Dementia Risk Score (DRS), QRISK3, with CVD as a proxy for both hearing loss and dementia risk	Piloting and further ­development of risk score tools for use, and integration into primary care or other health records
	Core20PLUS5	NHS England framework: focus on most deprived 20% and 5 key clinical areas to address health inequalities	Target the most deprived initially, then expand as feasible

PTA = pure tone audiometry, NHS = National Health Service, UKBDRS = UK Biobank Dementia Risk Score, CAIDE = cardiovascular risk factors, ageing, and dementia score, DRS = dementia risk score, QRISK3 = cardiovascular risk score 3, CVD = cardiovascular disease.

The NSC was established primarily to review the evidence for national universal population screening programs, and these are generally what have been commissioned through the NHS to date. With the appointment of a new chair in 2022 the value of targeted and stratified screening has been recognized and calls for such programs have been made. It is recognized that while a universal adult hearing screening program would perhaps meet population need more fully, a pragmatic way forward is to move beyond the binary of *universal versus no screening*. Targeted, risk-stratified hearing screening offers a middle ground. It focuses resources on early identification for those most likely to benefit (e.g., people at higher dementia risk, those with multiple hearing loss risk factors, and populations at greatest risk of health inequalities), while reducing the service burden that makes universal screening unattainable. This approach is aligned with the NHS prevention agenda, the Core20PLUS5 policy, and recent NSC signals that targeted screening initiatives should be considered alongside traditional universal models.

Current U.K. health policy provides a strong foundation for introducing targeted hearing screening among adults at high risk of dementia and therefore hearing loss, and those in deprived communities. NICE guidance recommends routine hearing assessment for individuals with dementia or mild cognitive impairment, with devolved nations like Wales, already integrating audiological assessment into dementia care pathways. The government’s “Fit for the Future: 10-Year Health Plan for England” emphasizes the need for prevention, community-based care, and digital innovation, naming dementia as a key priority for early diagnosis and intervention (Department of Health & Social Care, 2025).

Evidence indicates that hearing aids improve quality of life, mitigate dementia-related symptoms, and support independence, even if the precise causal impact on dementia incidence remains uncertain. The absence of definitive proof that hearing aids prevent dementia should not delay action. As we have argued, the shared risk factors, emerging and converging evidence, and consistent cost-effectiveness modeling, alongside evidence that delayed intervention results in poorer adaptation to hearing aids together provide a robust justification for large-scale piloting of targeted, risk-stratified screening.

Hearing screening tools are available, low cost, and can be delivered by trained non-specialist staff or via apps, with professional support and follow-up where needed. Adoption of existing sensitive screening tests would enable other, novel, low-touch options to be evaluated alongside them for continuous service delivery. Following any screen with a refer outcome, formal diagnostic assessment alongside a medical history would be obtained within audiology services enabling joint decisions to be made about the most appropriate care plan.

Operationally, consensus on eligibility criteria (i.e., the best combination of implementation pathway, risk scores, and screening tool(s) would need piloting, alongside the most appropriate settings for implementation such as in community pharmacy, primary care, or community diagnostic centers, which may vary by locality. Such pilots would allow feasibility, acceptability, and cost-effectiveness to be evaluated, while generating the evidence base required to inform larger-scale adoption and pathway development.

## Addressing concerns and limitations

No screening program is without risk. Potential harms include anxiety, stigma, or overdiagnosis, though for hearing these are limited compared to other screening initiatives. Table 3 outlines the criteria set by the UK NSC for targeted screening (The UK National Screening Committee, 2022). While evidence gaps remain around the impact of hearing aids on long-term ­dementia outcomes, delaying action risks widening inequities and lost opportunities for prevention. As with other public health measures, decisions must balance sufficient rather than perfect evidence in order to move forward and provide ­important pilot data.

**Table 3.** UK NSC targeted screening criteria and adult hearing screening (The UK National Screening Committee, 2022).

NSC targeted screening		
Criterion	Description	Meets criteria?
**1**	The health impact of the condition and its course should be understood, with evidence that serious disease can be identified or predicted by an agreed level of identifiable risk or marker.	Y
**2**	There should be evidence from appropriately designed studies and models on cost effectiveness for the screening test and intervention.	Y
**2 a.**	Screening test—this should be a simple test that has evidence of suitable accuracy and technical performance derived from studies in the population in which the test is being used	Y
**2 b.**	Intervention—there should be better outcomes from early intervention/those at a pre-symptomatic stage, for the screened individual, compared with usual care	Y
**3**	There should be a diagnostic investigation available for individuals with a positive screening test result, with evidence it can distinguish who would benefit from intervention from those who would not.	Y
**4**	The overall benefits from the screening programme should outweigh the harms (e.g., overdiagnosis, false positives, false reassurance and uncertain findings).	Y
**5**	There should be a robust and inclusive evidence-based selection criteria for identifying those eligible for targeted screening.	TBC
**6**	There should be evidence that the screening program is acceptable to the public and professionals, with appropriately balanced information available to those invited to attend screening.	TBC
**7**	The UK NSC should carry out a feasibility assessment (informed by NHS practice) that indicates the screening program would be achievable; with evidence for monitoring and quality assuring the program, adequate staffing and facilities being available to meet the requirements of program delivery.	TBC

TBC = to be confirmed, UK = United Kingdom, NSC = National Screening Committee, NHS = National Health Service.

## Policy call-to-action

The UK NSC’s traditional remit has focused on universal programs, but its openness to targeted and stratified models is promising. It is recognized that while a universal adult hearing screening program would perhaps meet the population need more fully than a targeted or risk stratified approach, these targeted initiatives would have less impact on the resources of the services offered by the NHS, meeting the needs for those at high risk of cognitive decline and/or hearing loss, while also supporting people from more disadvantaged backgrounds to seek help sooner.

The U.K. should conditionally adopt targeted and risk-stratified hearing screening following piloting in high-risk and -need populations, with rigorous evaluation of delivery models, patient experience and long-term outcomes. This recommended approach balances feasibility with equity, addresses unmet need, and positions the U.K. as a leader in shaping global hearing screening strategies.

We urge policymakers to

1. pilot targeted and risk stratified approaches to hearing screening using risk factors and risk scores, and starting in the most deprived communities;
2. evaluate cost-effectiveness and acceptability using real-world implementation studies;
3. engage diverse patients and carers in codesigning interventions to ensure relevance and accessibility to underserved groups; and
4. normalize hearing screening as part of healthy aging, reducing stigma and improving uptake.

## Conclusion

Hearing loss receives little attention considering it is a major modifiable driver of poor health and social outcomes. Whilst cost-effective, universal hearing screening in adults may be impractical in the U.K., but a targeted, risk-stratified strategy is both feasible and proportionate. Such an approach makes the best use of available resource, using sensitive and specific tests, is feasible and implementable, while creating a platform to validate emerging innovations that can transform accessibility for the wider population and reduce the resource burden. By focusing on those with the highest risk, we can reduce health inequalities, support those least likely to seek help, and generate the evidence needed for continuous service improvement. Although our case focuses on the U.K., the lessons are wider: in nations with constrained resources and persistent health inequalities, targeted, risk-stratified approaches offer a pragmatic alternative to universal programs, helping reduce inequities and providing models that other health systems can adapt. We therefore call upon policymakers to act now to gather the evidence through large scale pilots to address unmet need and promote equity.

## Data Availability

This article synthesizes evidence from previously published and publicly available sources. No new data were generated for this study. All data supporting the statements in this article are available in the article, cited references and public reports.
